# β-Amyloid is associated with aberrant metabolic connectivity in subjects with mild cognitive impairment

**DOI:** 10.1038/jcbfm.2014.66

**Published:** 2014-04-16

**Authors:** Felix Carbonell, Arnaud Charil, Alex P Zijdenbos, Alan C Evans, Barry J Bedell

**Affiliations:** 1 Biospective Inc., 6100 avenue Royalmount, Montreal, Quebec, Canada; 2 Montreal Neurological Institute, McGill University, Montreal, Quebec, Canada

**Keywords:** APOE *ɛ*4, *β*-amyloid, FDG PET, florbetapir PET, metabolic connectivity, mild cognitive impairment

## Abstract

Positron emission tomography (PET) studies using [18F]2-fluoro-2-deoxyglucose (FDG) have identified a well-defined pattern of glucose hypometabolism in Alzheimer's disease (AD). The assessment of the metabolic relationship among brain regions has the potential to provide unique information regarding the disease process. Previous studies of metabolic correlation patterns have demonstrated alterations in AD subjects relative to age-matched, healthy control subjects. The objective of this study was to examine the associations between *β*-amyloid, apolipoprotein E *ɛ*4 (APOE *ɛ*4) genotype, and metabolic correlations patterns in subjects diagnosed with mild cognitive impairment (MCI). Mild cognitive impairment subjects from the Alzheimer's Disease Neuroimaging Initiative (ADNI) study were categorized into *β*-amyloid-low and *β*-amyloid-high groups, based on quantitative analysis of [18F]florbetapir PET scans, and APOE *ɛ*4 non-carriers and carriers based on genotyping. We generated voxel-wise metabolic correlation strength maps across the entire cerebral cortex for each group, and, subsequently, performed a seed-based analysis. We found that the APOE *ɛ*4 genotype was closely related to regional glucose hypometabolism, while elevated, fibrillar *β*-amyloid burden was associated with specific derangements of the metabolic correlation patterns.

## Introduction

Regional glucose hypometabolism is a prominent feature of Alzheimer's disease (AD). [18F]2-fluoro-2-deoxyglucose (FDG) positron emission tomography (PET) has been extensively utilized in AD studies and characteristic patterns of AD-associated glucose hypometabolism have emerged.^1, 2^ The relationship between glucose hypometabolism and the underlying AD-associated neuropathological changes, such as accumulation of fibrillar *β*-amyloid plaques, however, remains poorly understood. In an early study, Mega *et al*^3^ found an inverse relationship between [18F]2-fluoro-2-deoxyglucose positron emission tomography (FDG PET) signal and biochemical measures of soluble and insoluble *β*-amyloid in prefrontal and parietooccipital cortical regions via co-registration of PET images and autopsy material from a single subject, thereby providing a basis for further investigations into the association between regional metabolic deficits and *β*-amyloid burden.

The recent advent of PET tracers for non-invasive visualization of *β*-amyloid deposits in human brain has dramatically improved our ability to explore potential relationships between amyloid burden and other disease biomarkers. Ikonomovic *et al*^4^ demonstrated a tight correlation between the retention of the amyloid PET tracer, Pittsburgh Compound B, with rigorous, region-matched analysis of *β*-amyloid plaques in a single subject. In a large cohort study, Clark *et al*^5^ firmly established the validity of [18F]florbetapir ([18F]AV-45, Amyvid) for assessment of amyloid burden via correlation of quantitative *in vivo* PET and postmortem neuropathology measures.

Mosconi and McHugh^2^ recently reviewed amyloid and FDG PET studies in AD, and discussed the synergies of these two techniques for improved early disease detection. Recently, Kadir *et al*^6^ found an increase in fibrillar amyloid load in mild cognitive impairment (MCI) patients followed by stabilization at the AD stage, while regional cerebral glucose metabolism declined in MCI patients and worsened with subsequent cognitive decline. In an intriguing study, Jagust and Landau^7^ reported that the apolipoprotein E *ɛ*4 (APOE *ɛ*4) genotype, and not aggregated fibrillar *β*-amyloid, contributes to glucose hypometabolism in cognitively normal, older subjects. While these studies have examined regional glucose metabolism, assessment of the metabolic correlations between regions has the potential to provide unique information regarding the association between *β*-amyloid and the functional network architecture in the AD brain.

In recent years, regional correlation analysis has been increasingly utilized for the study of the structural, functional, and metabolic connectivity architecture (i.e., connectome) of the brain. In a seminal paper, Horwitz *et al*^8^ described inter-subject metabolic correlations between the brain regions and identified strong correlations between homotopic regions and homologous regions in the left and right hemispheres. Through the application of this method to FDG PET data from AD subjects, this group found reduced interhemispheric and frontal–parietal correlations, suggesting a breakdown of organized functional activity.^9^ Mosconi *et al*^10^ examined the metabolic intercorrelations between the entorhinal cortex and the rest of the brain, and identified altered relationships between the entorhinal cortex and several cortical and limbic regions in AD. Several groups have recently extended this work to examine regional metabolic correlations across the entire brain based on FDG PET images from large-scale AD studies.^11, 12, 13^

The relationship between *β*-amyloid burden and metabolic correlations patterns, however, remains unexplored. To this end, we have performed a correlation analysis of FDG PET images from MCI subjects with low and high amyloid burdens. Given the recent observations of Jagust and Landau,^7^ we also assessed the effects of the APOE *ɛ*4 genotype on metabolic correlations. In this study, we employed a modified version of the MACACC (Mapping Anatomical Correlations across Cerebral Cortex) method reported by Lerch *et al*^14^ to obtain voxel-wise Metabolic Correlation Strength (MCS) maps across the entire cerebral cortex, which allowed us to examine differences in the cortical correlation structure between groups. We also performed a seed-based analysis to obtain a more detailed assessment of alterations in the cortical correlation architecture for specific cortical regions implicated in AD pathology.

## Materials and methods

### Subjects and Image Acquisition

Data used in the preparation of this article were obtained from the Alzheimer's Disease Neuroimaging Initiative (ADNI) database (adni.loni.ucla.edu). The ADNI was launched in 2003 by the National Institute on Aging, the National Institute of Biomedical Imaging and Bioengineering, the Food and Drug Administration, private pharmaceutical companies and non-profit organizations, as a $60 million, 5-year public private partnership. The primary goal of ADNI has been to test whether serial magnetic resonance imaging (MRI), PET, other biologic markers, and clinical and neuropsychological assessment can be combined to measure the progression of MCI and AD. Determination of sensitive and specific markers of very early AD progression is intended to aid researchers and clinicians to develop new treatments and monitor their effectiveness, as well as lessen the time and cost of clinical trials.

The Principal Investigator of this initiative is Michael W. Weiner, MD, VA Medical Center and University of California, San Francisco. Alzheimer's Disease Neuroimaging Initiative is the result of efforts of many co-investigators from a broad range of academic institutions and private corporations, and subjects have been recruited from over 50 sites across the United States and Canada. The initial goal of ADNI was to recruit 800 subjects but ADNI has been followed by ADNI-GO and ADNI-2. To date, these three protocols have recruited over 1,500 adults, ages 55 to 90, to participate in the research, consisting of cognitively normal older individuals, people with early or late MCI, and people with early AD. The follow-up duration of each group is specified in the protocols for ADNI-1, ADNI-2, and ADNI-GO. Subjects originally recruited for ADNI-1 and ADNI-GO had the option to be followed in ADNI-2. For up-to-date information, see www.adni-info.org.

The subjects of this report consisted of 276 ADNI participants diagnosed with MCI who had available [18F]florbetapir PET, FDG PET, 3D T1-weighted anatomic MRI, and APOE *ɛ*4 genotyping. A detailed description of the MRI and PET image acquisition protocols can be found at http://adni.loni.ucla.edu/about-data-samples/image-data. Alzheimer's Disease Neuroimaging Initiative studies are conducted in accordance with the Good Clinical Practice guidelines, the Declaration of Helsinki, and U.S. 21 CFR Part 50 (Protection of Human Subjects), and Part 56 (Institutional Review Boards). This study was approved by the Institutional Review Boards of all of the participating institutions. Informed written consent was obtained from all participants at each site.

### Image Processing

All MRI and PET images were processed using the PIANO software package (Biospective, Montreal, Canada). T1-weighted MRI volumes underwent image non-uniformity correction using the N3 algorithm, brain masking, linear spatial normalization utilizing a 9-parameter affine transformation, and nonlinear spatial normalization to map individual images from native coordinate space to MNI reference space using a customized, anatomic MRI template derived from ADNI subjects. The resulting image volumes were segmented into gray matter (GM), white matter, and cerebrospinal fluid using an artificial neural network classifier and partial volume estimation.^15^ The GM density map for each subject was transformed to the same final spatial resolution (i.e., re-sampled to the same voxel size and spatially smoothed) as the FDG PET data to account for confounding effects of atrophy in the statistical model. The cerebral mid-cortical surface (i.e., the mid-point between the pia and white matter) for each hemisphere was extracted to allow for surface projection of PET data using a modified version of the CLASP algorithm.^16^

The florbetapir PET and FDG images underwent several preprocessing steps, including frame-to-frame linear motion correction, smoothing using a scanner-specific blurring kernel, and concatenation of dynamic frames into a static image. The PET volumes were linearly registered to the subject T1-weighted MRI and, subsequently, spatially normalized to reference space using the nonlinear transformations derived from the anatomic MRI registration. Voxel-wise standardized uptake value ratio (SUVR) maps were generated from both florbetapir and FDG PET using full cerebellum and pons as the reference regions, respectively. The cortical SUVR measures were projected onto the cortical surface, and the data from each subject was mapped to a customized surface template by non-rigid 2D surface registration for visualization purposes.^17^

### Subject Classification

The mean [18F]florbetapir SUVR was computed from a composite bilateral region of interest comprising the precuneus, posterior cingulate, and medial frontal cortex, for each subject (SUVR_ROI_). A Regularized Discriminant Analysis (RDA)^18^ was performed to determine the optimal threshold to separate subjects into two distinct classes based on individual SUVR_ROI_ measurements. Regularized Discriminant Analysis (RDA) assumes an underlying Gaussian distribution and defines discriminative functions based on the sample means and covariance matrices. Regularized Discriminant Analysis (RDA) includes a regularization parameter that controls the degree of contraction of each individual class covariance matrix estimate (quadratic discriminant analysis) toward the pooled (over all classes) covariance matrix (linear discriminant analysis). As a result, RDA is a general discriminant analysis technique that includes linear discriminant analysis and quadratic discriminant analysis as particular cases.

For this study, individual [18]florbetapir SUVR_ROI_ measurements were ranked and cutoff values that separated measures into two different classes were defined. The RDA defined the contraction parameter that yielded the maximal accuracy at each cutoff. The optimal cutoff value of 1.22 for this data set was then determined via Receiver Operating Characteristic analysis. This optimal cutoff produced accuracy, specificity, and sensitivity values of 0.97, 0.99, and 0.96, respectively, based on the Receiver Operating Characteristic analysis. Subjects with an SUVR_ROI_ value⩽1.22 were designated as *β*-amyloid low (A*β*_L_), and this group consisted of 139 subjects with an average SUVR_ROI_ value of 1.03±0.08 (mean±s.d.). The remaining 137 subjects, with average SUVR_ROI_ values of 1.50±0.16, were classified as *β*-amyloid high (A*β*_H_). Further details of the subject characteristics are provided in Table 1.

### Subject Characteristic Analysis

An initial statistical analysis of subject characteristics was performed. The *β*-amyloid status (A*β*_L_ and A*β*_H_) and APOE *ɛ*4 genotype (non-carrier (APOE*ɛ*4_NC_) and carrier (APOE*ɛ*4_C_)) were treated as independent binary categorical variables. Cognitive performance measures, including the Mini-Mental State Exam (MMSE), the Alzheimer's Disease Assessment Scale-Cognitive Subscale, the Boston Naming Test, the Montreal Cognitive Assessment, and a composite score of executive function, were treated as continuous variables. The composite score of executive function is a composite score based on the ADNI-EF score described by Gibbons *et al.*^19^ In our case, we have utilized the Alternating Trail Making, Cube and Clock Drawing, Backward Digit Span, and Verbal Fluency sub-tests administered as part of the Montreal Cognitive Assessment assessment. Associations among categorical variables (e.g., gender, amyloid status, APOE *ɛ*4 genotype) were determined using contingency tables, while analysis of continuous variables (e.g., age, [18]florbetapir SUVR_ROI_, MMSE, Alzheimer's Disease Assessment Scale-Cognitive Subscale) was performed by analysis of variance (ANOVA). Specifically, sample size frequency distribution and the associations between gender and amyloid status or APOE *ɛ*4 genotype were analyzed with 2 × 2 contingency tables. A two-way ANOVA model that included [18]florbetapir SUVR_ROI_ as a dependent continuous variable, and amyloid status, APOE *ɛ*4, and amyloid × APOE *ɛ*4 as predictors of interest, was assessed. Analogous two-way ANOVAs were also performed using age, MMSE, Alzheimer's Disease Assessment Scale-Cognitive Subscale, Boston Naming Test, Montreal Cognitive Assessment, and composite score of executive function as continuous dependent variables. The statistical significance for all tests was set at *α*=0.05. All values are reported as mean±s.d.

### Voxel-wise Analysis of [18F]Florbetapir Standardized Uptake Value Ratio, [18F]2-fluoro-2-deoxyglucose Standardized Uptake Value Ratio, and Gray Matter Density

The influence of amyloid status and APOE *ɛ*4 genotype on [18F]florbetapir SUVR data were analyzed by a voxel-wise approach. A two-way analysis of covariance model that included [18F]florbetapir SUVR as dependent variable, age, gender, and MMSE as covariates, and amyloid status, APOE *ɛ*4 genotype, and amyloid status × APOE *ɛ*4 interaction as predictors of interest was assessed. An analogous analysis of covariance model was also used to investigate the influence of amyloid status and APOE *ɛ*4 genotype on GM density maps. A similar, voxel-wise, two-way analysis of covariance model was fitted to the FDG SUVR data. This analysis of covariance design included age, gender, and MMSE as global covariates, as well as GM density as a voxel-wise covariate, which minimized potential confounds related to GM volume.^20, 21^ The predictors of interest in this model were amyloid status, APOE *ɛ*4, and amyloid status × APOE *ɛ*4 interaction. *Post hoc*, two-tailed Student's *t*-tests were performed to assess the main effects of interest and interaction terms.

The voxel-wise statistical analysis was performed using the SurfStat toolbox (http://www.math.mcgill.ca/keith/surfstat). The t-statistic maps corresponding to each main effect of interest were thresholded using the false-discovery rate procedure (*α*=0.05) to control for multiple comparisons.^22^

### Metabolic Correlation Strength Analysis

We performed an analysis of MCS to assess the overall pattern of metabolic connectivity across the cerebral cortex in each group. This novel, exploratory approach also allows for unbiased identification of cortical hubs. The basic principle of MCS is to determine the extent to which the FDG SUVR at any particular voxel is correlated with the SUVR of all other cortical voxels.

Our approach for determination of MCS measures is a modified version of the MACACC strength measure employed by Lerch *et al*^14^ to assess the structural correlations based on cortical thickness data. As originally defined by Lerch *et al*,^14^ the MACACC strength at any voxel is defined as the average of the Pearson's correlation coefficients between that voxel and all other voxels in the cortex. In our particular implementation, we performed a Fisher's Z-transformation of the correlation coefficients before entering them into the averaging process. This modification allows for proper averaging of the individual correlations to compute the MCS value, which is critical given that non-transformed Pearson's correlation coefficients are not strictly additive metrics. The MCS maps were constructed as follows:

1. Compute Pearson's correlation coefficient (*r*) between each pair of voxels.
2. Map all cross-correlations into a Gaussian scale (Z-scale) by means of Fisher's Z-transformation, *Z(r)=arctanh(r).*
3. For each voxel, compute the average of the corresponding Z-transformed cross-correlations to all other voxels.
4. Compute the inverse of Fisher's Z-transformation to re-define the MCS on the original correlation coefficient scale.

Given the highly summarizing nature of the MCS measures, it is generally not appropriate to assess statistically significant differences between groups. Rather, the MCS maps serve as a useful exploratory tool for revealing within-group highly correlated regions (i.e., MCS hubs). In this study, we identified local maxima (defined as one s.d. above the mean of all voxels) as MCS hubs and generated seed-based correlation maps at each of these hubs. These seed-based correlation maps demonstrate the metabolic connectivity pattern associated with a particular hub and allow for statistical interrogation of group differences.

### Seed-Based Metabolic Correlation Analysis

The ‘seed region' consisted of a spherical region of interest with a 6-mm radius centered at each hub. For every subject, the average FDG SUVR values were computed within each seed region. Seed-based cross-correlation maps were generated by correlating the mean seed region FDG SUVR value with all other voxels over the entire cortex for each group.^23^ The between-group comparisons (A*β*_L_ versus A*β*_H_; APOE*ɛ*4_NC_ versus APOE*ɛ*4_C_) of corresponding seed-based correlation maps were performed by generating a voxel-wise Z-statistic map using the formula:^24^


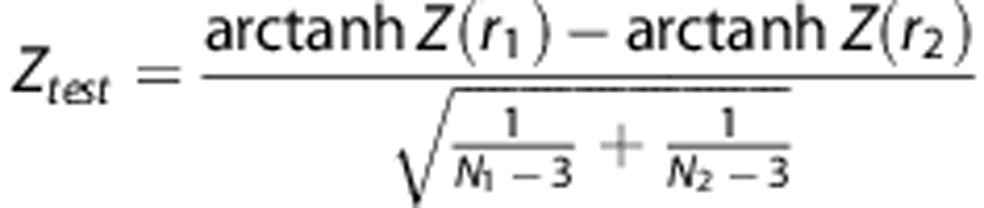


where *r*_*1*_ and *r*_*2*_ denote the seed-based cross-correlation maps, and *N*_*1*_ and *N*_*2*_ are the number subjects in the two groups under comparison. Statistically significant between-group seed-based correlations were determined by thresholding the Z-statistic map using a false discovery rate threshold of *α*=0.05.

## Results

### Subject Characteristic Analysis

Subject characteristics are reported in Table 1. Of the 139 A*β*_L_ subjects, 35 (25.2%) were APOE *ɛ*4 carriers, while 98 of 137 A*β*_H_ subjects (71.5%) were APOE *ɛ*4 carriers. This sample size distribution produced a significant association between amyloid status and APOE *ɛ*4 genotype (*χ*^2^=57.55, *P*<0.001). Although the whole sample was not equally distributed between males and females, the association between gender and amyloid status was not statistically significant (*P*=0.79), nor was the association between gender and APOE *ɛ*4 genotype (*P*=0.32).

The two-way ANOVA model with SUVR_ROI_ as dependent variable confirmed that the [18F]florbetapir SUVR_ROI_ values of the A*β*_H_ subjects (1.50±0.16) were significantly higher than those of the A*β*_L_ subjects (1.03±0.08) (*P*<0.001). In contrast, there was no statistically significant effect of the APOE *ɛ*4 genotype on the [18F]florbetapir SUVR_ROI_ values (*P*=0.33). Interrogation of the interaction terms in the two-way ANOVA model showed that APOE *ɛ*4 genotype does not have a significant effect in any of the groups (*P*=0.11 for A*β*_L_; *P*=0.09 for A*β*_H_).

The two-way ANOVA with age as dependent variable revealed that the age of A*β*_H_ subjects (74.22±7.02) was significantly higher than that of A*β*_L_ subjects (71.47±8.60) (*P*<0.001). The main effect of APOE *ɛ*4 genotype on age also demonstrated statistically significant differences. Specifically, the APOE*ɛ*4_NC_ subjects (74.33±8.16) were significantly older than the APOE*ɛ*4_C_ subjects (71.20±7.43) (*P*<0.001). The ANOVA with MMSE as dependent variable revealed a statistically significant main effect of amyloid status (*P*=0.02), but no main effect of APOE *ɛ*4 genotype (*P*=0.43), on this measure. There was also a statistically significant main effect of amyloid status (*P*<0.001), but no significant main effect of APOE *ɛ*4 genotype (*P*=0.33), on Alzheimer's Disease Assessment Scale-Cognitive Subscale. Likewise, the ANOVA with the Boston Naming Test produced a statistically significant main effect of amyloid status (*P*=0.007), but no main effect of APOE *ɛ*4 genotype (*P*=0.25). For the Montreal Cognitive Assessment score, the *P*-values corresponding to the main effects of amyloid status and APOE *ɛ*4 genotype were 0.04 and 0.08, respectively. However, there were no main effects of amyloid status (*P*=0.48) or APOE *ɛ*4 genotype (*P*=0.14) on the composite score of executive function score.

### [18F]Florbetapir Standardized Uptake Value Ratio, [18f]2-fluoro-2-deoxyglucose Standardized Uptake Value Ratio, and Gray Matter Density Analysis

**Figure 1:**
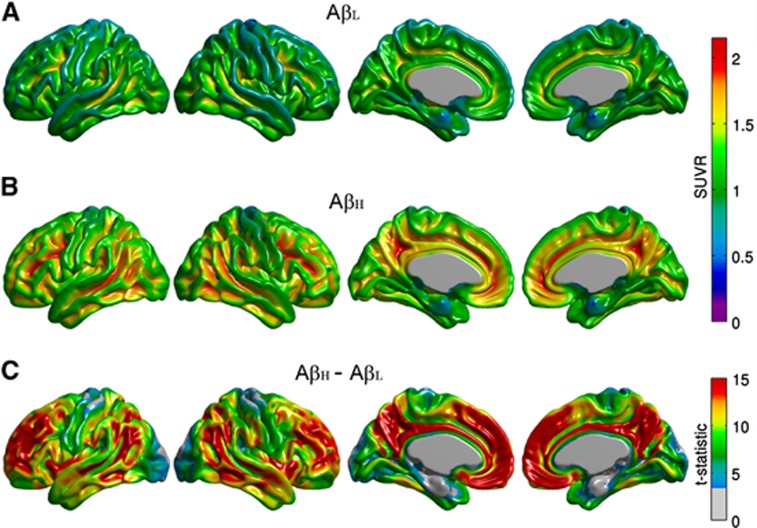
Representative surface views (left-to-right: left lateral, right lateral, left medial, and right medial) of the average florbetapir standardized uptake value ratio (SUVR) maps for the *β*-amyloid low (A*β*_L_) (**A**) and *β*-amyloid high (A*β*_H_) groups (**B**). Note the negligible tracer binding in the A*β*_L_ group in contrast to the elevated regional levels of florbetapir uptake in the cerebral cortex in the A*β*_H_ group, as demonstrated by the t-statistic maps (**C**).

The average [18]florbetapir SUVR maps for the A*β*_L_ and A*β*_H_ groups are shown in Figure 1. The A*β*_H_ group showed significantly higher levels of cortical uptake, relative to the A*β*_L_ group, in a number of bilateral cortical regions, including the cingulate gyrus, precuneus, parietotemporal cortex, and frontal regions. This distribution recapitulates the [11C]Pittsburgh Compound B binding pattern in AD subjects previously reported by Buckner *et al.*^25^

The false discovery rate-thresholded t-statistic maps corresponding to the main effects of amyloid status and APOE *ɛ*4 genotype on GM density are shown in Supplementary Figure 1. The A*β*_L_ group showed significantly greater GM density than the A*β*_H_ group, particularly in the lateral temporal and inferior parietal regions. In contrast, the main effect of APOE *ɛ*4 genotype did not reveal marked group differences. Similar results were obtained when GM density was replaced by GM volume.

**Figure 2:**
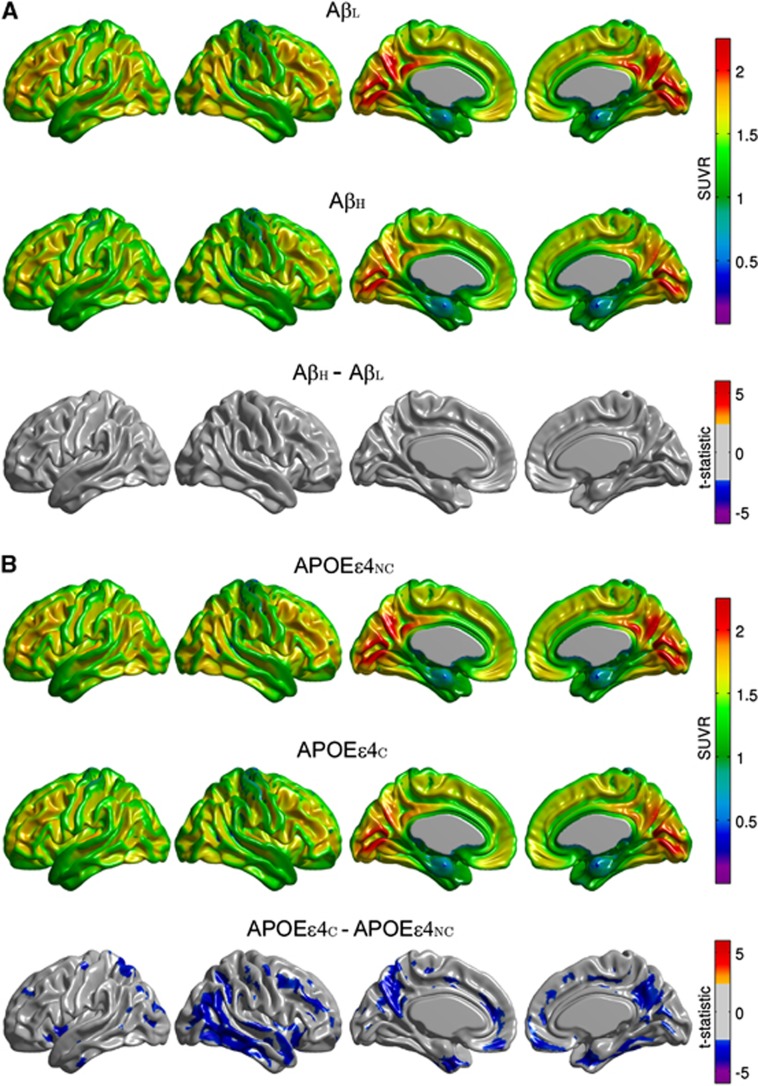
Representative surface views of the average [18F]2-fluoro-2-deoxyglucose (FDG) standardized uptake value ratio (SUVR) maps for *β*-amyloid low (A*β*_L_) and *β*-amyloid high (A*β*_H_) groups, and thresholded (*α*=0.05) t-statistic maps for the A*β*_H_ versus A*β*_L_ differences (**A**). Average FDG SUVR maps for the APOE*ɛ*4_NC_ and APOE*ɛ*4_C_ groups, and corresponding t-statistic maps for the group differences (**B**). Note the relative absence of significant group differences between the A*β*_L_ and A*β*_H_ groups, while the APOE*ɛ*4_C_ group shows significant regional hypometabolism relative to the APOE*ɛ*4_NC_ group.

The main effect of amyloid status on FDG SUVR did not reveal statistically significant group differences (Figure 2A). In contrast, the main effect of APOE *ɛ*4 genotype showed significantly reduced regional glucose metabolism in the APOE*ɛ*4_C_ group, particularly in the right lateral temporal and parietal lobes, bilaterally in the posterior cingulate/precuneus, entorhinal cortex, and regions of the medial frontal cortex (Figure 2B). Additional exploration of the interaction term effects revealed that the significant differences between the APOE*ɛ*4_NC_ and APOE*ɛ*4_C_ groups largely originated from the A*β*_H_ group (Supplementary Figure 2).

### Metabolic Correlation Strength Analysis

**Figure 3:**
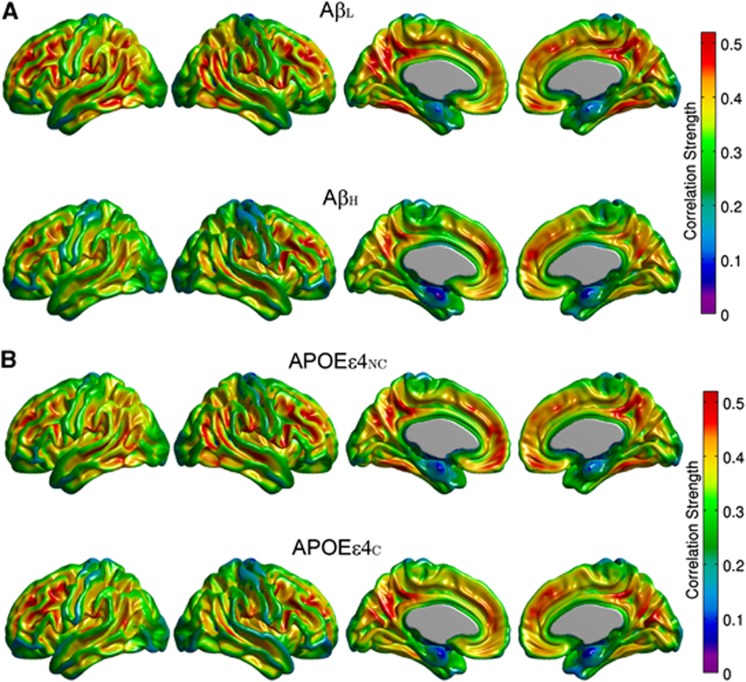
Metabolic Correlation Strength (MCS) maps based on amyloid status (**A**) and apolipoprotein E *ɛ*4 (APOE *ɛ*4) genotype (**B**). Note that the highly correlated (hub) regions (e.g., angular, supramarginal, inferior temporal gyri) are conserved across groups. The *β*-amyloid high (A*β*_H_) group demonstrates widespread reductions in MCS compared with the *β*-amyloid low (A*β*_L_) group, most notably in the paracentral lobule, lateral parietal cortex, and inferior temporal cortex, whereas the APOE*ɛ*4_NC_ and APOE*ɛ*4_C_ groups show similar MCS patterns.

The FDG MCS maps for the A*β*_L_ and A*β*_H_ groups are shown in Figure 3A, while the maps for the APOE*ɛ*4_NC_ and APOE*ɛ*4_C_ groups are provided in Figure 3B. The A*β*_L_ group showed regions of strong correlations, bilaterally, in the precuneus/posterior cingulate gyrus, inferior parietal (supramarginal and angular gyri), medial frontal cortex, pars opercularis, and fusiform gyrus. It is interesting to note that the regions with the strongest correlations are similar to those with high amyloid deposition in the A*β*_H_ group (Figure 1B). While the highly summarizing nature of the MCS measure does not readily lend itself to detection of statistically significant group differences, reduced regional MCS is clearly apparent in the A*β*_H_ group, particularly in the lateral parietal cortex, inferior temporal cortex, and paracentral lobule. In contrast, differences between APOE*ɛ*4_NC_ and APOE*ɛ*4_C_ groups are not as prominent. This data suggest that increased amyloid burden is associated with a reduction of metabolic correlations. In order to further interrogate this effect, seed-based correlation analysis was performed using the cortical hubs revealed by the MCS maps, specifically precuneus, fusiform gyrus, pars opercularis, supramarginal and angular gyri, inferior temporal gyrus, medial frontal cortex, and paracentral lobule, as seed regions.

### Seed-Based Metabolic Correlation Analysis

**Figure 4:**
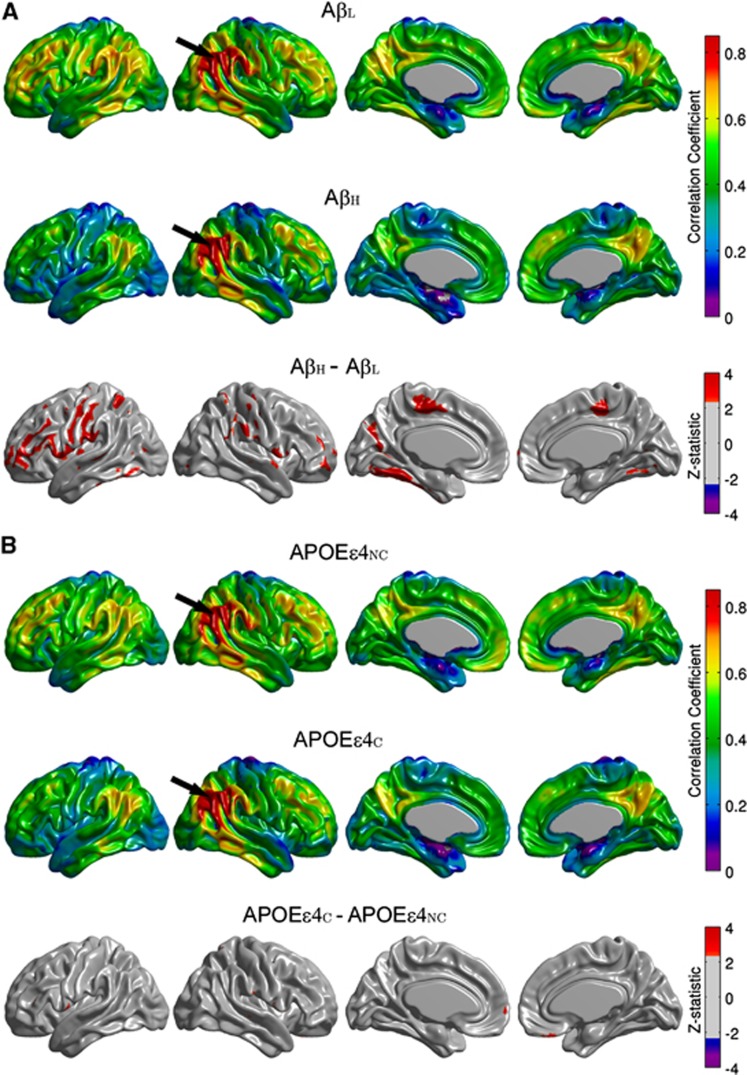
Seed-based correlation maps for the right angular gyrus. Maps are provided for the *β*-amyloid low (A*β*_L_) group, *β*-amyloid high (A*β*_H_) group, and thresholded Z-statistic for the A*β*_L_ versus A*β*_H_ group differences (**A**). The arrows indicate the seed region. Note the significantly reduced correlations in the A*β*_H_ group, especially with the left fusiform gyri, bilateral paracentral lobule, bilateral inferior frontal gyrus, and left precentral and postcentral gyri. Seed-based correlation maps for apolipoprotein E *ɛ*4 noncarrier (APOE*ɛ*4_NC_) group, apolipoprotein E *ɛ*4 carrier (APOE*ɛ*4_C_) group, and thresholded Z-statistic for the APOE*ɛ*4_NC_ versus APOE*ɛ*4_C_ group differences are shown in (**B**).

Between-group comparisons of the seed-based correlation maps demonstrated that only the angular gyrus, inferior temporal gyrus, paracentral lobule, and supramarginal gyrus hubs yielded statistically significant differences with substantial spatial extent. The seed-based correlation maps for the right angular gyrus seed, along with the false discovery rate-thresholded Z-statistic parametric map, are shown in Figure 4. The A*β*_H_ group showed reduced intra- and interhemispheric correlations compared with the A*β*_L_ group, especially between the seed and the left fusiform gyri, bilateral paracentral lobule, bilateral inferior frontal gyrus (pars opercularis), and left precentral and postcentral gyri (Figure 4A). The correlation coefficient between the left and right angular seeds was also reduced (*P*=0.017) in the A*β*_H_ group (*r*=0.66) compared with the A*β*_L_ group (*r*=0.78) (Table 2). With the exception of a few, scattered, significant clusters that showed increased correlations (medial frontal and bilateral pars opercularis regions) in the APOE*ɛ*4_NC_ group, the differences between APOE*ɛ*4_NC_ and APOE*ɛ*4_C_ groups (Figure 4B) were small in relation to the A*β*_L_ versus A*β*_H_ comparisons.

**Figure 5:**
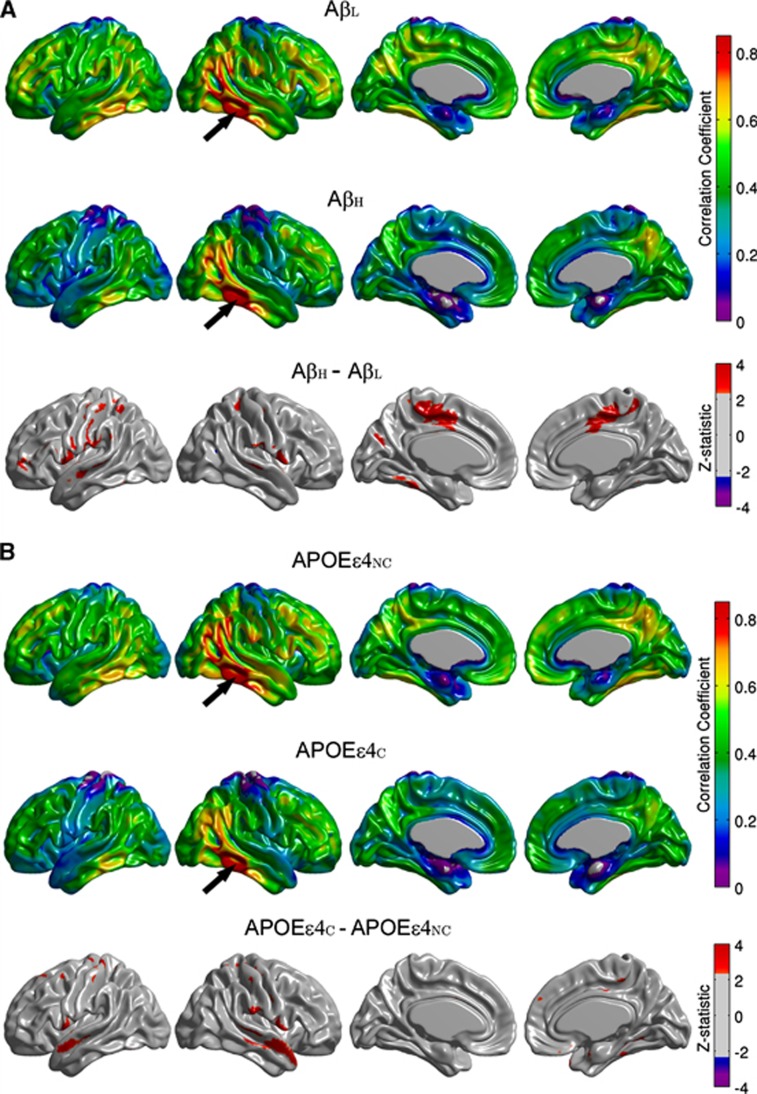
Seed-based correlation maps for the right inferior temporal gyrus. Maps are provided for the *β*-amyloid low (A*β*_L_) group, *β*-amyloid high (A*β*_H_) group, and thresholded Z-statistic for the A*β*_L_ versus A*β*_H_ group differences (**A**). The arrows indicate the seed region. Note the significantly reduced correlations A*β*_H_ group, especially with the bilateral paracentral lobule, supramarginal gyrus and pars opercularis. Maps for apolipoprotein E *ɛ*4 noncarrier (APOE*ɛ*4_NC_) group, apolipoprotein E *ɛ*4 carrier (APOE*ɛ*4_C_) group, and thresholded Z-statistic of the APOE*ɛ*4_NC_ versus APOE*ɛ*4_C_ group differences are provided in (**B**). Some significantly reduced correlations are evident between the seed and the superior temporal gyrus in the APOE*ɛ*4_C_ group.

The seed-based correlation analysis corresponding to the right inferior temporal gyrus seed showed reduced correlations in the A*β*_H_ group, especially between the seed and bilateral areas of the paracentral lobule, supramarginal gyrus, pars opercularis, and left fusiform gyrus (Figure 5A). The correlation coefficient between the left and right inferior temporal gyrus seeds was also statistically significantly reduced (*P*=0.037) in the A*β*_H_ group (*r*=0.68) compared with the A*β*_L_ group (*r*=0.78) (Table 2). Consistent with the observations for the right angular gyrus seed, the statistically significant differences between the APOE*ɛ*4_NC_ and APOE*ɛ*4_C_ groups (Figure 5B) appear to be highly localized. In particular, correlations between the right inferior temporal gyrus and the bilateral superior temporal gyrus showed significant reductions in the APOE*ɛ*4_C_ group relative to the APOE*ɛ*4_NC_ group.

Supplementary Figure 3 shows the seed-based correlation maps for the left supramarginal gyrus seed, as well as thresholded Z-statistic maps of the main effects of amyloid status and APOE *ɛ*4 genotype. The A*β*_H_ group showed reduced metabolic correlations relative to the A*β*_L_ group, especially between the seed and the right lateral parietal cortex and postcentral gyrus (Supplementary Figure 3A). Although not statistically significant, the correlation coefficient between the left and right supramarginal gyrus seeds was also reduced (*P*=0.069) in the A*β*_H_ group (*r*=0.74) compared with the A*β*_L_ group (*r*=0.81) (Table 2).

The seed-based correlation analysis corresponding to the left paracentral lobule seed showed reduced correlations in the A*β*_H_ group, especially between the seed and bilateral areas of the inferior temporal gyrus and lateral parietal cortex, as well as the right fusiform gyrus (Supplementary Figure 4A). The correlation coefficient between the left and right paracentral lobule seeds was reduced (*P*=0.027) in the A*β*_H_ group (*r*=0.84) compared with the A*β*_L_ group (*r*=0.89) (Table 2). While correlations between the left paracentral lobule and the right inferior temporal gyrus showed significant reductions in the APOE*ɛ*4_C_ group relative to the APOE*ɛ*4_NC_ group, the differences between the APOE*ɛ*4_NC_ and APOE*ɛ*4_C_ groups (Supplementary Figure 4B) were less extensive than those between the A*β*_L_ and A*β*_H_ groups.

Table 2 summarizes the main effects of amyloid status and APOE *ɛ*4 genotype on the correlation coefficients between homologous seeds in the left and right hemispheres. This table includes all seeds revealed as hubs by the MCS maps, as well as a seed in the entorhinal cortex. Although not classified as a hub in the MCS maps, the entorhinal cortex is known to be affected early in AD pathogenesis and it has been shown to demonstrate AD-related alterations in metabolic correlations.^1^ There is an evident amyloid-related decrease in interhemispheric metabolic correlations between homologous regions in the A*β*_H_ group relative to the A*β*_L_ group in all seed regions. In contrast, there is no evident main effect of APOE *ɛ*4 genotype on the interhemispheric correlations with the exception of the paracentral lobule.

## Discussion

In this work, we have explored the associations between metabolic correlation, fibrillar *β*-amyloid burden, and APOE *ɛ*4 genotype in MCI subjects from the ADNI study. Our results of conventional SUVR-based group comparisons of FDG PET data are in agreement with those of Jagust and Landau,^7^ demonstrating that regional glucose hypometabolism appears to be intimately linked with the APOE *ɛ*4 genotype in both cognitively intact and MCI subjects. However, our MCS maps and seed-based correlation analysis indicate that alterations of the ‘metabolic connectome' are related to the presence of fibrillar, *β*-amyloid deposits and are not a function of genotype. A potential explanation to reconcile this double dissociation is that the APOE *ɛ*4 genotype confers susceptibility to ‘coordinated glucose hypometabolism', presumably reflecting neurodegeneration, whereas high *β*-amyloid levels are associated with ‘metabolic heterogeneity'. The latter may be a consequence of variable spatiotemporal patterns of compensatory responses and/or cortical remodeling during the preclinical/early stages of AD.

Our approach employed a statistical model-based GM density correction^20, 21^ of the SUVR data. Inclusion of the individual GM density maps as a voxel-wise covariate in our analysis of the FDG SUVR data effectively minimized potential confounds related to GM differences associated with amyloid status. Based on our VBM analysis, we found a statistically significant reduction of GM density values in the A*β*_H_ subjects relative to the A*β*_L_ group, particularly in the lateral temporal and inferior parietal regions. As such, in contrast to regional glucose hypometabolism, which appears to be intimately linked with the APOE *ɛ*4 genotype, GM atrophy has a stronger association with *β*-amyloid burden. Our results agree with the reported discordant topography between atrophy and hypometabolism in amnestic MCI patients.^26^ In fact, our results suggest that such a topographical mismatch may be explained by APOE *ɛ*4 genotype.

Previous metabolic connectivity analysis has typically relied on either predefined seeds of interest or region of interest-based analysis. Mosconi *et al*^10^ proposed a voxel-wise seed-based correlation analysis within the entorhinal cortex. Lee *et al*^23^ systematically explored the whole brain metabolic correlation pattern of healthy young adults with anatomically predefined seeds of interest. Alternative approaches of assessing metabolic connectivity are based on a whole brain, region of interest-based correlation analysis.^27, 28^ Morbelli *et al*^29^ indicated that patterns of metabolic connectivity strongly depend on the anatomic locations of the seeds when employing systematic seed-based correlation analysis. In this work, we have introduced the MCS measure, which provides a data-driven means of identifying hub regions for systematic seed-based metabolic correlation analysis. The MCS maps (Figure 3) show a spatial pattern of high metabolic correlation that is remarkably similar to *β*-amyloid deposition (Figure 1B). Our MCS maps derived from FDG PET images are also similar to connectivity maps derived from resting-state blood oxygenation level-dependent functional MRI,^25^ despite the fact that the former are generated from MCI patients, while the latter were produced from young, healthy subjects. As suggested by Buckner *et al*,^25^ the topography of hubs may explain the pattern of regional vulnerability in AD.

More specifically, as cortical hubs are regions of high intrinsic activity and metabolism associated with information processing (e.g., heteromodal association areas, default-mode network), they could possess accelerated AD-related pathology.^25^ Our MCS analysis revealed metabolic cortical hubs that have previously been shown to demonstrate reduced activity and functional disconnection in AD, and are associated with increased amyloid burden.^25, 30^ We assessed metabolic correlations related to two hubs located in the inferior parietal cortex, namely the angular gyrus (Figure 4) and the supramarginal gyrus (Supplementary Figure 3). Our results agree with those ones reported by Jacobs *et al*,^31^ which implicated strong connectivity patterns between the parietal lobe and other brain areas as a driving factor for the involvement of the parietal lobe in AD. In particular, the angular gyrus mediates language and semantic processing, as well as spatial attention and orientation, and is a key parietal node of the default-mode network.^32^ The inferior temporal gyrus has an important role in mediating verbal fluency, a cognitive function that is affected early in AD.^33^ It has been shown that *β*-amyloid deposition in the inferior temporal neocortex is also strongly related to hippocampal synaptic and neuronal degeneration.^34^ This relationship has been explained by the well-established, AD-related, disruption of connectivity between the inferior temporal cortex and the hippocampus.^35^ Our results suggest that the disruption of metabolic correlations between the inferior temporal gyrus and several brain areas (Figure 5) appear to also be associated with *β*-amyloid burden. Regardless of APOE *ɛ*4 genotype status, the cognitive decline at early stages of AD has recently been linked to the *β*-amyloid deposition in several regions, including the paracentral lobule.^36^ Our results also suggest the metabolic connectivity of the paracentral lobule is modulated by *β*-amyloid irrespective of the APOE *ɛ*4 genotype.

In contrast to many other PET studies, which have primarily focused on healthy control (HC) and/or AD subjects, our study population consisted of MCI subjects. This population tends to demonstrate heterogeneity in various cognitive performance metrics and progression to AD. We have shown that the A*β*_L_ MCI group demonstrated less cognitive impairment than the A*β*_H_ MCI group, except with respect to executive function. Interestingly, no statistically significant differences as a function of APOE *ɛ*4 genotype were observed with respect to the *β*-amyloid deposition or cognitive performance in this group of MCI subjects.

In addition to the unique observations of our study, we identified several features in our comparisons of A*β*_L_ and A*β*_H_ MCI groups that were similar to those of previous investigations, which focused on differences in interregional metabolic correlation between age-matched HC and AD subjects. Mosconi *et al*^10^ noted reduced correlations between the entorhinal cortex and the ipsilateral temporal–parietal cortex, as well as between the entorhinal cortex and the contralateral hemisphere, with the exception of the homologous region, in AD subjects. Morbelli *et al*^11^ performed a seed-based, voxel-wise correlation analysis of FDG PET data in prodromal AD and age-matched HC subjects from the European Alzheimer Disease Consortium project, and found disease-related alterations in hypometabolic areas, hippocampus, and dorsolateral frontal cortex seed regions. This group suggested that reduced metabolic correlation (i.e., disconnection) in both hypometabolic and non-hypometabolic regions in prodromal AD subjects may precede remote hypometabolism (reflecting synaptic degeneration). Based on our study, it appears that this early disconnection phenomenon is strongly associated with *β*-amyloid deposition.

In an elegant study, Drzezga *et al*^30^ explored the relationship between resting-state blood oxygenation level dependent-derived functional connectivity, FDG PET, and amyloid PET measures in amyloid-positive and -negative HC subjects, as well as amyloid-positive MCI subjects. This group identified a significant disruption of MRI-derived functional whole-brain connectivity in cortical hub regions (posterior cingulate/precuneus and temporal–parietal cortex) in amyloid-positive MCI subjects. They also observed overlap between these connectivity disruptions and regional hypometabolism. Further, they found that increased amyloid burden was correlated with reduced whole-brain connectivity and glucose metabolism, especially in the posterior cingulate cortex/precuneus hub region. Our data comparing A*β*_L_ and A*β*_H_ MCI subjects lend further support to the vulnerability of hub regions to *β*-amyloid accumulation and functional/metabolic disconnection.

The decreased cross-correlations between homologous regions in the left and right hemispheres in the A*β*_H_ group are particularly interesting. Bero *et al*^37^ performed a functional connectivity analysis, based on optical intrinsic signal data from a mouse model of AD at the early stages of disease, and found reduced correlations between homologous cerebral cortical regions. They also identified a strong relationship between these regional bilateral connectivity measures and regional susceptibility to *β*-amyloid deposition. It is intriguing that the reduced bilateral functional correlations in mice and metabolic correlations in humans are strongly related to *β*-amyloid. While the establishment of a concrete relationship requires additional investigation, this data support the potential for effective translatability of results across species and imaging modalities. Future studies could examine the metabolic correlation structure in AD mouse models using FDG microPET or quantitative autoradiography studies. Further, given that correspondence between resting-state functional magnetic resonance imaging and FDG PET^12^ and arterial spin labeling perfusion MRI, and FDG PET^38^ have been demonstrated, comparisons of the respective correlation structures between modalities could be pursued.

When put in the context of previous functional/metabolic correlation studies in AD, our results support the notion of a progressive deterioration and reorganization of the cortical correlation architecture as a function of the underlying pathologic processes. Seeley *et al*^39^ have proposed several mechanistic hypotheses to explain brain network-based disease patterns in neurodegenerative diseases, including nodal stress, transneuronal spread, trophic failure, and shared vulnerability.^40^ As indicated by this group, molecular pathology approaches may help to clarify nature of regional vulnerability to the disease process. Drzezga *et al*^30^ have also postulated several mechanisms to relate regional neuronal loss, synaptic dysfunction, energy consumption, and functional connectivity. The demonstration of compromised functional connectivity in a transgenic mouse model of AD by Bero *et al*^37^ opens the possibility to interrogate the precise relationship between imaging and neuropathological measures, which is not typically feasible in human studies. A thorough understanding of the cellular and molecular basis of altered functional/metabolic correlation patterns in AD would facilitate the use of these measures as valid, clinically translatable, non-invasive biomarkers for early diagnosis/prognosis and evaluation of the efficacy of putative disease-modifying therapeutic agents.

## Figures and Tables

**Table 1:** Summary of subject characteristics

	*All subjects*	*Aβ*_*L*_	*Aβ*_*H*_	*APOEɛ4*_*C*_	*APOEɛ4*_*NC*_
Sample size	276	139	137	132	144
SUVR_ROI_	1.26±0.27	1.03±0.08	1.50±0.16	1.38±0.24	1.16±0.24
Age	72.83±7.96	71.47±8.60	74.22±7.02	71.20±7.43	74.33±8.16
Gender (F/M)	119/157	61/78	58/79	61/71	58/86
MMSE	27.34±3.25	28.22±2.44	26.45±3.71	26.93±3.68	27.71±2.76
ADAS-Cog	16.47±9.44	12.84±6.57	20.18±10.46	18.48±10.69	14.64±7.73
BNT	26.67±3.55	27.35±3.01	25.96±3.93	26.84±3.86	26.48±3.25
MoCA	23.99±4.25	24.17±4.35	23.81±4.14	24.40±4.28	23.61±4.19
CompEF	5.52±1.45	5.57±1.45	5.47±1.47	5.63±1.43	5.42±1.48

A*β*_*H*_, *β*-amyloid high; A*β*_*L*_, *β*-amyloid low, ADAS-Cog, Alzheimer's Disease Assessment Scale-Cognitive Subscale; APOE*ɛ*4_*C*_, apolipoprotein E *ɛ*4 carrier; APOE*ɛ*4_*NC*_, apolipoprotein E *ɛ*4 noncarrier; BNT, Boston Naming Test; CompEF, composite score of executive function; MMSE, Mini-Mental State Exam; MoCA, Montreal Cognitive Assessment; ROI, region of interest; SUVR, standardized uptake value ratio.

**Table 2:** Interhemispheric Pearson's correlation coefficients between homologous seeds and *P*-values for group differences

*Seeds*	*Aβ*_*L*_	*Aβ*_*H*_	P*-value (Aβ*_*L*_*-Aβ*_*H*_)	*APOEɛ4*_*C*_	*APOEɛ4*_*NC*_	P*-value (APOEɛ4*_*C*_*-APOEɛ4*_*NC*_)
ANG	0.78	0.66	0.017	0.72	0.73	0.451
ENT	0.68	0.57	0.052	0.66	0.60	0.183
FUSI	0.86	0.84	0.237	0.86	0.83	0.174
ITG	0.78	0.68	0.037	0.75	0.70	0.210
MFC	0.89	0.87	0.242	0.89	0.86	0.137
OPER	0.78	0.69	0.037	0.82	0.76	0.071
PCL	0.89	0.84	0.027	0.90	0.84	0.026
PHG	0.72	0.58	0.027	0.69	0.62	0.148
PRECUN	0.92	0.91	0.387	0.90	0.93	0.094
SMG	0.81	0.74	0.069	0.76	0.80	0.192

A*β*_*H*_, *β*-amyloid high; A*β*_*L*_, *β*-amyloid low; ANG, angular gyrus; APOE*ɛ*4_*C*_, apolipoprotein E *ɛ*4 carrier; APOE*ɛ*4_*NC*_, apolipoprotein E *ɛ*4 noncarrier; ENT, entorhinal cortex; FUSI, fusiform gyrus; ITG, inferior temporal gyrus; MFC, medial frontal cortex; OPER, pars opercularis; PCL, paracentral lobule; PHG, parahippocampal gyrus; PRECUN, precuneus; SMG, supramarginal gyrus.

## APPENDIX

Data used in preparation of this article were obtained from the Alzheimer's Disease Neuroimaging Initiative (ADNI) database (adni.loni.ucla.edu). As such, the investigators within the ADNI contributed to the design and implementation of ADNI and/or provided data but did not participate in the analysis or writing of this report. A complete listing of ADNI investigators can be found at: http://adni.loni.ucla.edu/wp-content/uploads/how_to_apply/ADNI_Acknowledgement_List.pdf.

## Notes

FC, AC, and APZ are employees of Biospective. APZ has received shares from Biospective. ACE and BJB have received compensation and shares from Biospective.
