# Supplementary material for: β-Amyloid is associated with aberrant metabolic connectivity in subjects with mild cognitive impairment
Source: J Cereb Blood Flow Metab. 2014 Apr 16;34(7):1169–79. doi: 10.1038/jcbfm.2014.66 (PMC4083380; doi:10.1038/jcbfm.2014.66)
Supplement: Supplementary Figures [file jcbfm201466x1.pdf]

Supplementary Figures

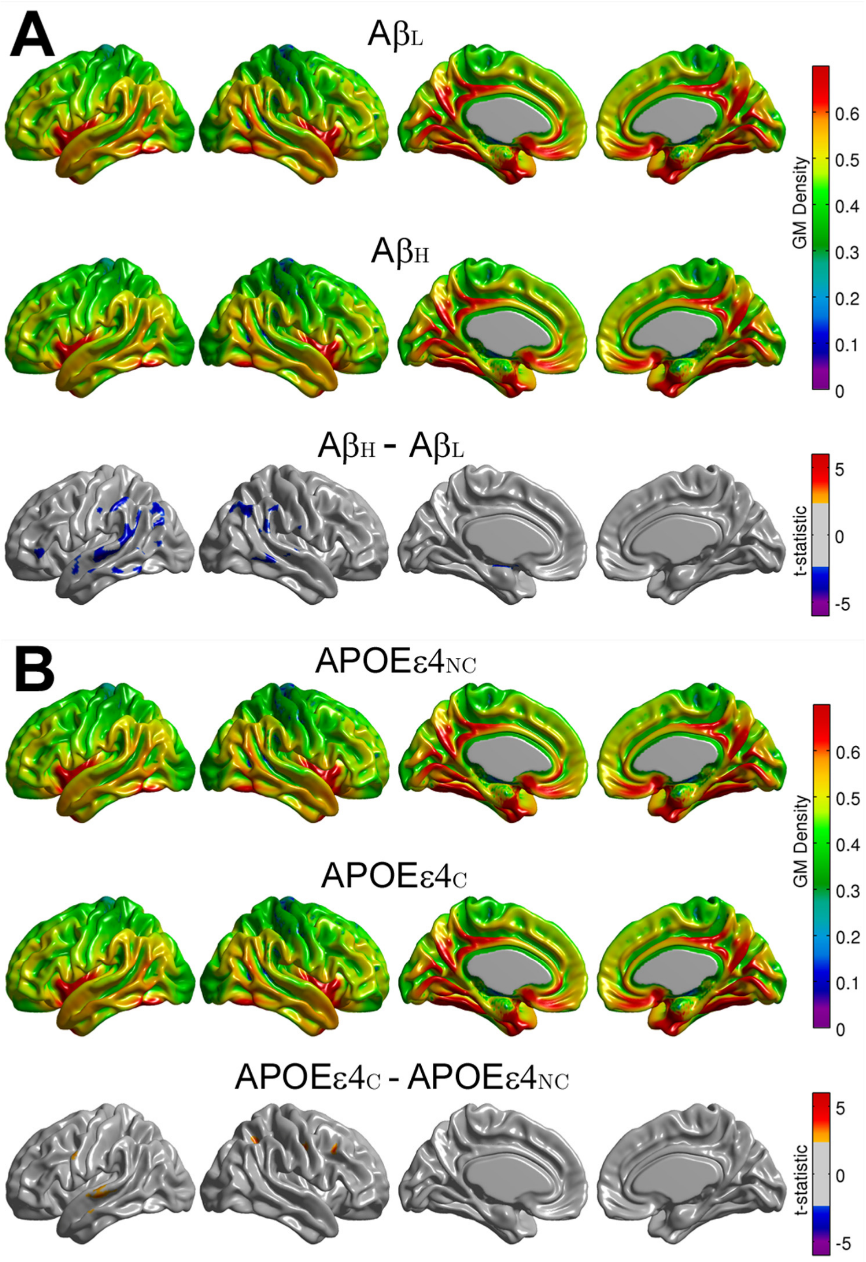

Supplementary Figure 1. Representative surface views of the GM density maps and thresholded ($\alpha = 0.05$) t-statistic maps corresponding to the main effects of amyloid status and APOE ε4 genotype on the gray matter density. The $A\beta_H$ group showed significantly reduced GM density in the lateral temporal and inferior parietal regions (A). The main effect of APOE ε4 genotype did not reveal marked group differences (B).

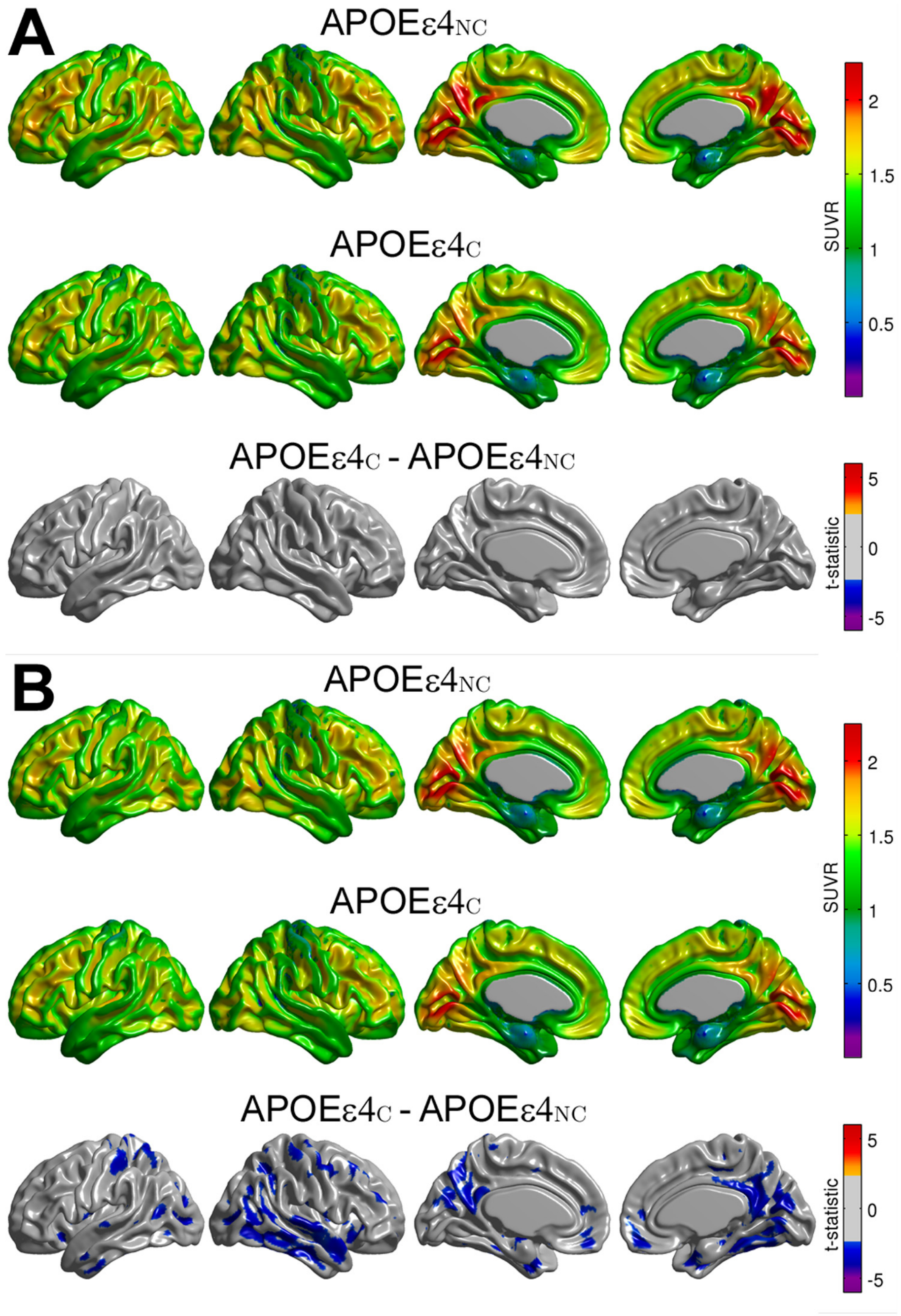

Supplementary Figure 2. Statistical comparison between the APOEε4$_{NC}$ and APOEε4E4$_{C}$ groups corresponding to Aβ$_{L}$ (A) and Aβ$_{H}$ (B) groups (*i.e.* ANOVA interaction terms). Note the absence of significant APOE ε4-related differences in the Aβ$_{L}$ group, while the APOEε4$_{C}$ group shows significant regional hypometabolism relative to the APOEε4$_{NC}$ group in the Aβ$_{H}$ group interaction term.

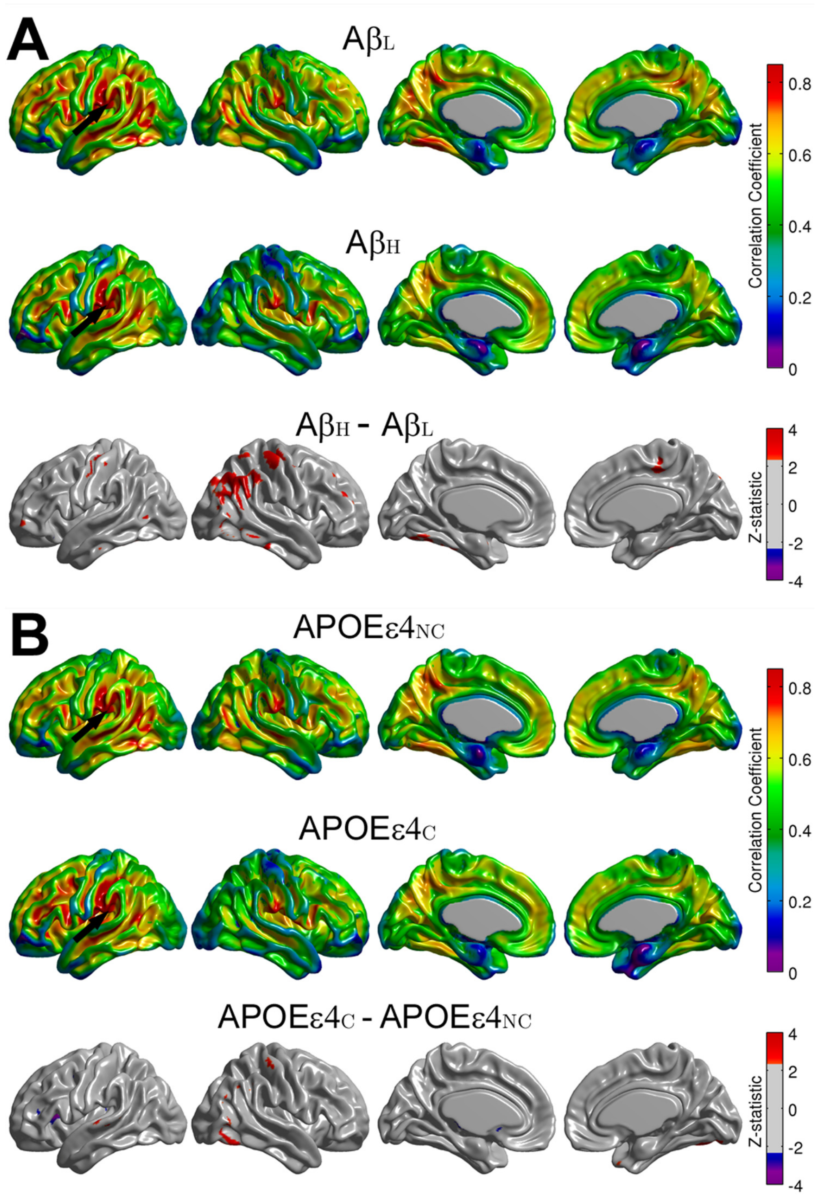

Supplementary Figure 3. Seed-based correlation maps for the Left Suprmarginal Gyrus. Maps are provided for the Aβ_L group, Aβ_H group, and thresholded Z-statistic for the Aβ_L vs. Aβ_H group differences (A). The arrows indicate the seed region. The Aβ_H group showed reduced correlations relative to the A$_L$ group, especially between the seed and the right lateral parietal lobe. Seed-based correlation maps for APOE$\varepsilon 4_{NC}$ group, APOE$\varepsilon 4_C$ group, and thresholded Z-statistic for the APOE$\varepsilon 4_{NC}$ vs. APOE$\varepsilon 4_C$ group differences are shown in (B). Only a few small clusters showing statistically significant differences are observable.

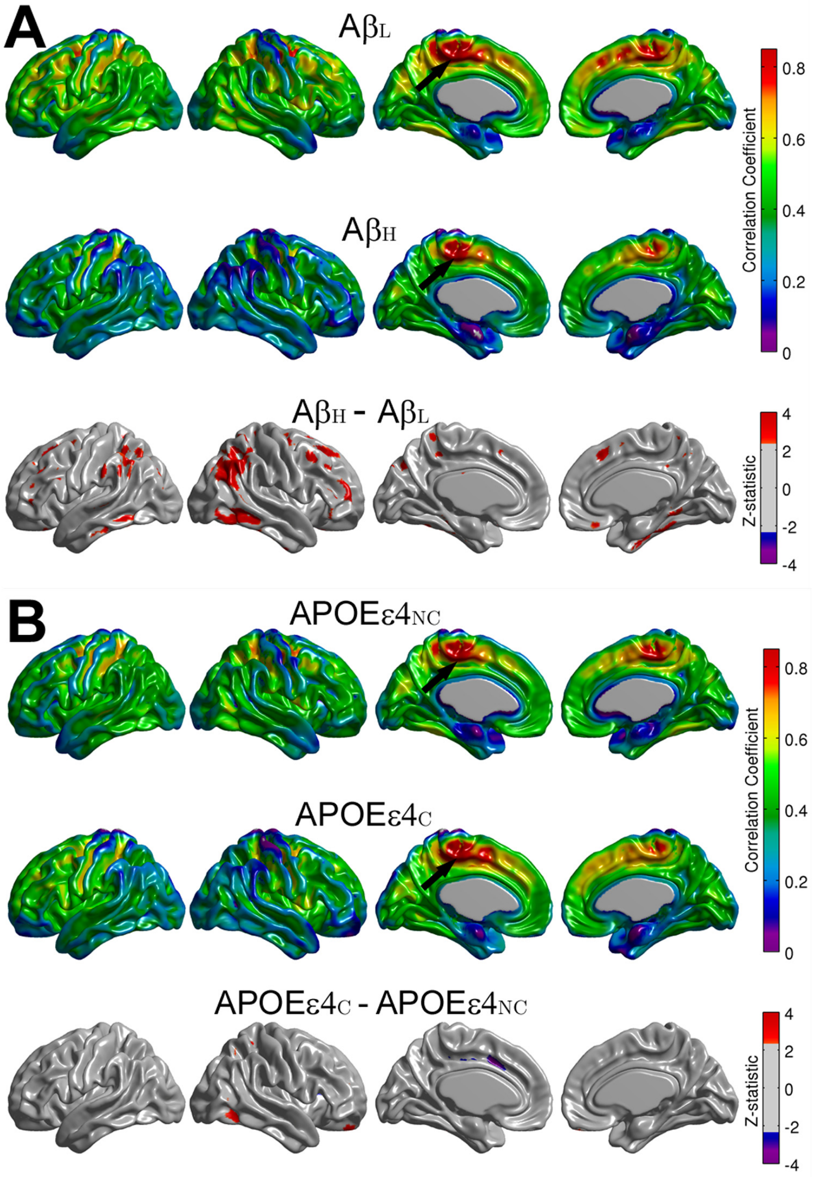

Supplementary Figure 4. Seed-based correlation maps for the Left Paracentral Lobule. Maps are provided for the $A\beta_L$ group, $A\beta_H$ group, and thresholded Z-statistic for the $A\beta_L$ vs. $A\beta_H$ group differences (A). The arrows indicate the seed region. The $A\beta_H$ group showed reduced correlations relative to the A_L group, especially between the seed and the inferior temporal gyrus and lateral parietal lobe. Maps for $APOE\varepsilon4_{NC}$ group, $APOE\varepsilon4_C$ group, and thresholded Z-statistic of the $APOE\varepsilon4_{NC}$ vs. $APOE\varepsilon4_C$ group differences are provided in (B). Only a few small clusters showing statistically significant differences between the $APOE\varepsilon4_{NC}$ and $APOE\varepsilon4_C$ groups.
